# Fructosamine Is a Useful Indicator of Hyperglycaemia and Glucose Control in Clinical and Epidemiological Studies – Cross-Sectional and Longitudinal Experience from the AMORIS Cohort

**DOI:** 10.1371/journal.pone.0111463

**Published:** 2014-10-29

**Authors:** Håkan Malmström, Göran Walldius, Valdemar Grill, Ingmar Jungner, Soffia Gudbjörnsdottir, Niklas Hammar

**Affiliations:** 1 Unit of Epidemiology, Institute of Environmental Medicine, Karolinska Institutet, Stockholm, Sweden; 2 Unit of Cardiovascular Epidemiology, Institute of Environmental Medicine, Karolinska Institutet, Stockholm, Sweden; 3 Department of Cancer Research and Molecular Medicine, Norwegian University of Science and Technology, Trondheim, Norway; 4 Department of Endocrinology, Trondheim University Hospital, Trondheim, Norway; 5 CALAB Research, Stockholm, Sweden; 6 Department of Medicine, Sahlgrenska University Hospital, University of Gothenburg, Gothenburg, Sweden; 7 Department of Epidemiology, AstraZeneca R&D, Mölndal, Sweden; University of Catanzaro Magna Graecia, Italy

## Abstract

**Context:**

Fructosamine is a glycemic biomarker which may be useful for indication and control of diabetes respectively.

**Objective:**

The objective of the study was to evaluate fructosamine as an indicator of hyperglycaemia and glucose control in subjects with diabetes.

**Design, Setting & Patients:**

From the AMORIS cohort, subjects with serum glucose, fructosamine and HbA_1c_ from the same examination were studied cross-sectionally and longitudinally (n = 10,987; 5,590 overnight-fasting). The guidelines of the American Diabetes Association were followed for classification of prediabetes and diabetes. Separate analyses were performed in patients with a newly detected or a known diagnosis of type 1 or type 2 diabetes respectively.

**Results:**

All three biomarkers were strongly correlated. With regard to the association between fructosamine and HbA_1c_ Pearson linear correlation coefficients in the range of 0.67–0.75 were observed in fasting and non-fasting subjects with type 1 or type 2 diabetes. Analyses of glucose control in fasting patients with type 2 diabetes having all three biomarkers measured at three separate occasions within on average 290 days of the index examination showed similar trends over time for glucose, fructosamine and HbA_1c_. Discrimination of subjects with and without diabetes across the range of fructosamine levels was good (area under curve (AUC) 0.91–0.95) and a fructosamine level of 2.5 mmol/L classified subjects to diabetes with a sensitivity of 61% and a specificity of 97%.

**Conclusions:**

Fructosamine is closely associated with HbA_1c_ and glucose respectively and may be a useful biomarker of hyperglycaemia and glucose control in clinical and epidemiological studies.

## Introduction

Fasting plasma glucose (FPG) and hemoglobin A_1c_ (HbA_1c_) are established indicators of glucose status and its control. Definitions from different health organizations use FPG or HbA_1c_ cut-offs of ≥7 mmol/L and ≥6.5% (48 mmol/mol) respectively either as single indicators or combined to diagnose diabetes [1]–[5]. HbA_1c_ reflects the glucose concentration during the entire lifespan of the red blood cells but to the largest extent the 6–8 weeks preceding the time of measurement [6]. Certain factors, e.g. red blood cell disorders, could potentially bias the measurement of HbA_1c_ and therefore alternative markers could be useful [6]. One such marker is fructosamine which relates to average levels of glucose during the preceding 1 to 3 weeks [6], [7]. Fructosamine may give an earlier indication of poorly controlled glucose compared to HbA_1c_. It is a simple, robust and inexpensive biomarker that could potentially be a useful tool in large epidemiological and clinical studies either as a standalone indicator of hyperglycaemia or in combination with glucose and HbA_1c_ [8]. Importantly, fructosamine may be reliably measured irrespective of fasting or non-fasting. However, fructosamine is rarely used in clinical practice and has not been extensively evaluated as an indicator of diabetes and its micro-and macro vascular complications in large population based studies. Various cut-offs for fructosamine as well as correlations to glucose and HbA_1c_ have been published based on rather small cohorts of patients [9]–[14]. Recently, fructosamine has been showed to be an independent biomarker to predict incident diabetes and its microvascular complications [15].

The aim of the present study was to explore the relationship between serum glucose, serum fructosamine and HbA_1c_ cross-sectionally and over time in a large population with a wide variation in glucose and HbA_1c_ values. An additional aim was to investigate the sensitivity and specificity of various fructosamine cut-offs in identification of subjects with prediabetes and diabetes using established diagnostic criteria based on HbA_1c_ and FPG.

## Materials and Methods

The study was based on the AMORIS cohort that has been described in detail previously [16], [17]. This cohort was recruited during 1985–1996 mainly from the greater Stockholm area. The cohort subjects were healthy and referred for clinical laboratory testing as part of a health check-up or were outpatients referred for laboratory testing. No samples were from inpatients. The AMORIS population consists of 812,073 subjects of all ages with varying information on a number of biomedical factors. Subjects were frequently followed with repeated examinations. For a total of 10,987 individuals, 5,199 males and 5,788 females, there was complete in­formation on serum glucose, serum fructosamine and HbA_1c_ from the same ex­amination. Of these individuals, 5,590 were known to be overnight-fasting at the time of the index examination which was defined as the first time glucose, fructosamine and HbA_1c_ were measured at the same occasion. For a repeated measurement analysis we identified type 2 diabetes patients (n = 254) with assessments of all biomarkers at the index examination as well as within 0–6 months and 6–12 months. In the case of multiple assessments within one of these time windows, we used the assessment farthest from the index examination. Subjects were grouped on whether they had increased or reduced glucose levels at the third examination.

### Laboratory methods

All laboratory examinations were performed at the CALAB laboratory (CALAB laboratories, Stockholm, Sweden), a laboratory accredited by SWEDAC (Swedish Board for Technical Accreditation). Serum glucose was measured enzymatically with a glucose oxidase/peroxidase method based on an enzymatic colorimetric technique according to the GOD-PAP method with automated multichannel analyzers (AutoChemist-PRISMA (PRISMA) and Technicon DAX 96). Total imprecision was CV<3%. Values of serum glucose are virtually identical to plasma glucose only differing by about 1% [18]. In the AMORIS study serum glucose values were standardized versus international standards of plasma glucose.

Fructosamine was measured by the Nitroblue Teterazolium (NBT) colorimetric technique devised by Johnson [19] based on the reducing ability of fructosamine in an alkaline solution and performed with the same automated analyzers as for serum glucose. Total imprecision was CV<5%.

Two methods were used to determine HbA_1c_. From 1992 and until the end of 1994 the method was based on affinity binding and quantitative determination of glycated hemoglobin in anticoagulated whole blood [20], [21] and performed with the Abbot Vision System. The results were ´adjusted to be HbA_1c_ and calibrated to DCCT values. Total CV was 4.4% at level 4.8 mmol/L and 2.8% at level 8.9 mmol/L. Reagents and three levels of calibrators from Abbott Diagnostics (Lake Forest, Illinois, USA) were used. The second method used since 1995 was based on a two-channel method, UNIMATE [22], automated on the analyzer Hitachi 917, for the immunological/colorimetric determination of % HbA_1c_. Here HbA_1c_ was measured by latex enhanced turbidimetry [23] and total Hb colorimetrically [24]. Total CV was <4.5%.

Albumin was colorimetrically measured based on its binding with bromocresol green. The CV was ≤2.0% at level 35 and 46 g/L and ≤2.5% at level 26 g/L. The concentration of albumin could potentially influence the concentration of fructosamine [7]. Hence, when possible we also calculated albumin corrected fructosamine using the following formula: (fructosamine (mmol/L)/albumin (g/L) x100) [25]. Glomerular filtration rate (eGFR) was estimated by the Epidemiology Collaboration (CKD-EPI) formula [26]. Classification of chronic kidney disease was defined as an eGFR less than 60 mL/min per 1.73 m^2^.

### Classification of glucose tolerance

We used the plasma glucose levels and HbA_1c_ values as defined by the American Diabetes Association (ADA) guidelines [1], [2] for the diagnosis of type 2 diabetes. Subjects were classified into five groups: normal glucose tolerance, prediabetes, newly diagnosed type 2 diabetes (NewT2D), previously diagnosed type 2 diabetes (DiagnosedT2D) and type 1 diabetes (T1D). This classification was based on glucose level at the index examination or by information of type 1 or type 2 diabetes prior to index examination based on other sources. Following the guidelines of ADA, we classified fasting serum glucose (FSG) below 5.6 mmol/L or HbA_1c_ below 5.7% (39 mmol/mol) as normal glucose tolerance. FSG of 5.6–6.9 mmol/L or HbA_1c_ between 5.7% and 6.4% (39–46 mmol/mol) was classified as prediabetes. FSG above 6.9 mmol/L or HbA_1c_ of 6.5% (48 mmol/mol) and above was classified as type 2 diabetes (NewT2D). In addition, if a subject had a random glucose of 11.1 mmol/L or above this was considered as NewT2D. Subjects who had information about hospitalization with diabetes as discharge diagnosis prior to the index examination were classified according to the discharge diagnosis (DiagnosedT2D). Subjects registered in the Swedish National Diabetes Register (NDR) were classified as type 1 (T1D) or type 2 diabetes (DiagnosedT2D) according to the diagnostic criteria set out in the register. As regards NDR, if no specific type was defined the clinical diagnosis by the referring physician was used. When registered as unspecified diabetes in the NDR or detected by the glucose criterion in AMORIS we classified subjects less than 30 years of age as T1D. Subjects diagnosed by the Swedish medical birth register (MFR) with gestational diabetes within one year prior or one year after the index examination were excluded from this study.

### Patient characteristics

Socioeconomic factors were obtained from several databases maintained by the Statistics Sweden. Those databases were the1990 national education database, the 1990 national census for occupation and the multi-generation register to obtain country of birth. Information on medical history including cardi­ovascular events, overall anemia, nephrosis and cancer prior to the index examination were obtained from inpatient registers and the cancer register maintained by the Swedish National Board of Health and Welfare. BMI was collected from the AMORIS laboratory database when assessed at any examination or from the NDR, national survey of living conditions (ULF, Statistics Sweden), the medical birth register (MFR, National Board of Health and Welfare), the Swedish Twin Registry or quality of care registers included in SWEDEHEART (Uppsala Clinical Research Center, UCR). The BMI closest to the date of the index examination was used for adjustment of the associations. Data on BMI were available in about 40% of subjects. Serum albumin, creatinine, lipoproteins and apolipoproteins were obtained from the index examination. Triglycerides, total cholesterol, albumin and creatinine were available for about 90% and high and- low density lipoprotein cholesterol (HDL-C, LDL-C) as well as apolipoproteins A-1 and B (ApoA-1, ApoB) for about 50% of the subjects.

### Ethical statement

This study is part of a project entitled “Metabolic abnormalities and inflammation in relation to chronic disease primarily cardiovascular diseases, kidney disease, cancer, dementia, rheumatoid arthritis and psychiatric illness – epidemiological studies based on the AMORIS population”.

This project has been approved by the Regional Ethical committee at Karolinska Institutet, Stockholm, Sweden (Registration number 2010/1∶7). All the study data was de-identified and the Ethical committee approved the project without collecting any additional informed consent.

### Statistical Analysis

All analyses were performed using SAS software (SAS Institute Inc., Cary, NC, USA). Pearson’s correlation coefficients and partial correlations were calculated using the SAS correlation procedure. A change in the point estimate of 10% or more was used as criteria for inclusion of a covariate in the multivariable models for confounding adjustment purposes. Sensitivity and specificity to classify diabetes were calculated using binary tables at various levels of fructosamine and with ADA criteria as the reference standard. Receiving operator characteristic analyses (ROC) were performed and area under the curve (AUC) with 95% Wald confidence limits estimated according to Mann-Whitney with PROC LOGISTIC. Series of the arithmetic mean values for glucose and HbA_1c_ per percentile of fructosamine were graphically displayed with corresponding 1 SD range using PROC MEANS and PROC SGPLOT. For the repeated measurement analyses, arithmetic mean values by biomarker and time window were calculated and graphically presented.

## Results

### Characteristics of subjects

Subject characteristics are presented in Table 1. There were 5,714 subjects (51% of all) who were cat­egorized as having normal glucose tolerance, 1,877 with prediabetes (17%) and 3,116 with type 2 diabetes (29%). Of the subjects classified as type 2 diabetes, 1,256 were diagnosed at the index examination i.e. NewT2D and 1,860 had a known diagnosis of type 2 diabetes. A total of 280 patients were diagnosed with type 1 diabetes. Fasting subjects in the NewT2D group (n = 759) were detected by a glucose value of 7 mmol/l or above in 94% of the subjects. Simultaneously, 51% of those subjects also had HbA_1c_ above 6.5%. The gender distribution was balanced, 47% males and 53% females, although females were relatively fewer among subjects with type 2 diabetes.

**Table 1:** Subject characteristics by glucose and fasting status.

	Normal		Prediabetes		NewT2D		DiagnosedT2D		T1D		
	Fasting	Non-fast	Fasting	Non-fast	Fasting	Non-fast	Fasting	Non-fast	Fasting	Non-fast	ALL
**N**	2,558	3,156	1,360	517	759	497	825	1,035	88	192	10,987
**% of pop**	23%	28%	12%	5%	7%	5%	8%	9%	1%	2%	100%
**Female sex**	66%	60%	52%	44%	33%	35%	37%	41%	44%	46%	53%
**Age (mean)**	56 (17)	55 (17)	61 (13)	62 (14)	61 (12)	62 (12)	60(11)	62 (11)	41 (11)	40 (13)	57 (16)
**Age (years)**											
** -30**	206	294	14	7	0	0	1	1	19	46	588
** 30–49**	720	925	252	95	131	73	151	155	47	93	2,642
** 50–69**	950	1,217	690	241	432	273	514	600	22	52	4,991
** 70-**	682	720	404	174	196	151	159	279	0	1	2,766
BMI*	24 (4.7)	26 (4.9)	27 (4.7)	28 (4.9)	29 (4.7)	29 (5.3)	29 (5.0)	28 (5.0)	26 (4.6)	26 (4.3)	27 (5.1)
**S-Fructosamine** †	2.03 (0.18)	2.05 (0.18)	2.11 (0.21)	2.24 (0.26)	2.62 (0.52)	2.83 (0.54)	2.71 (0.58)	2.77 (0.55)	2.93 (0.62)	3.11 (0.63)	2.28 (0.48)
**B-HbA1c**	4.76 (0.42)	4.86 (0.39)	5.14 (0.53)	5.84 (0.40)	6.93 (1.66)	7.92 (1.53)	7.21 (1.77)	7.41 (1.70)	7.62 (1.58)	7.88 (1.56)	5.69 (1.52)
**S-Glucose**	4.92 (0.41)	5.23 (0.80)	5.99 (0.47)	6.97 (1.54)	9.55 (3.01)	11.1 (4.20)	9.61 (3.60)	10.5 (4.60)	11.3 (5.33)	10.5 (5.73)	6.87 (3.28)
**cFructosamine**	4.72 (0.46)	4.72 (0.47)	4.92 (0.53)	5.14 (0.60)	6.18 (1.25)	6.65 (1.33)	6.44 (1.46)	6.50 (1.34)	6.83 (1.27)	7.34 (1.49)	5.29 (1.16)
**S-Alb**	43.0 (2.7)	43.5 (2.9)	43.0 (2.6)	43.4 (2.8)	42.9 (2.6)	42.7 (2.9)	42.7 (3.1)	42.8 (2.9)	42.3 (3.4)	42.9 (3.2)	43.1 (2.8)
**S-TG**	1.25 (0.81)	1.52 (1.12)	1.69 (1.33)	2.03 (1.14)	2.30 (1.80)	2.56 (2.43)	2.32 (2.30)	2.24 (1.98)	1.61 (1.31)	1.27 (0.91)	1.71 (1.48)
**S-TC**	5.75 (1.16)	5.76 (1.19)	5.99 (1.15)	6.08 (1.15)	6.07 (1.20)	5.98 (1.26)	6.01 (1.47)	5.96 (1.37)	5.45 (1.32)	5.42 (1.48)	5.86 (1.24)
S-LDLC*	3.58 (1.02)	3.63 (1.05)	3.79 (1.01)	3.91 (1.06)	3.83 (1.15)	3.80 (1.07)	3.71 (1.14)	3.71 (1.13)	3.36 (1.28)	3.31 (1.15)	3.67 (1.06)
S-HDLC*	1.61 (0.41)	1.52 (0.43)	1.48 (0.40)	1.36 (0.37)	1.27 (0.44)	1.23 (0.39)	1.32 (0.45)	1.30 (0.40)	1.58 (0.58)	1.60 (0.43)	1.48 (0.43)
ApoB*	1.14 (0.33)	1.19 (0.36)	1.28 (0.35)	1.33 (0.37)	1.40 (0.41)	1.40 (0.48)	1.39 (0.48)	1.31 (0.42)	1.27 (0.61)	1.04 (0.37)	1.23 (0.38)
ApoA-I*	1.46 (0.23)	1.44 (0.23)	1.44 (0.20)	1.41 (0.19)	1.38 (0.23)	1.38 (0.24)	1.39 (0.24)	1.38 (0.21)	1.46 (0.26)	1.46 (0.23)	1.43 (0.22)
ApoB/ApoA*	0.80 (0.26)	0.85 (0.28)	0.91 (0.28)	0.96 (0.26)	1.04 (0.34)	1.03 (0.31)	1.03 (0.40)	0.97 (0.31)	0.88 (0.41)	0.73 (0.27)	0.88 (0.30)
**S-Crea**	77.2 (16.8)	80.2 (24.0)	82.1 (18.5)	85.8 (16.8)	85.7 (16.3)	86.7 (19.0)	86.4 (19.7)	89.4 (35.7)	85.2 (23.8)	82.2 (18.7)	81.9 (22.2)
**eGFR^§^**	84.5 (18.4)	83.8 (18.8)	79.2 (16.9)	76.1 (17.3)	79.3 (16.3)	77.5 (17.2)	79.3 (17.1)	76.0 (19.4)	91.8 (20.3)	92.4 (17.8)	81.7 (18.4)
**Education** ‡	24%	29%	28%	34%	35%	35%	37%	36%	20%	21%	30%
**Sweden born**	79%	82%	80%	80%	79%	76%	77%	81%	86%	90%	80%
**B-C, Workers**	10%	14%	12%	13%	14%	13%	18%	17%	19%	17%	14%
**History, CVD**	4%	5%	6%	6%	7%	6%	13%	14%	7%	3%	7%
**History, cancer**	8%	8%	10%	10%	7%	8%	7%	8%	2%	3%	8%
**CKD** £	9%	11%	14%	18%	14%	16%	12%	21%	9%	7%	12%
**Anemia**	1.4%	1.5%	1.5%	1.6%	0.9%	0.2%	1.8%	2.5%	2.3%	1.0%	1.5%

Data are presented as n, mean (SD) or %. *on average 50–60% reduction in n.

† Significant difference between groups, except between NewT2D and DiagnosedT2D,

‡ % only mandatory education.^§^Glomerular filtration rate (eGFR) was estimated by the Epidemiology Collaboration (CKD-EPI) formula (26).

£ Classification of chronic kidney disease was defined as an eGFR less than 60 mL/min per 1.73 m^2^. Abbreviations: BMI = Body Mass Index, S-Alb = Serum Albumin, S-TG = Serum Triglycerides, S-TC = Serum Total Cholesterol, S-LDLC = Serum Low Density Lipoprotein Cholesterol, S-HDLC = Serum Low Density Lipoprotein Cholesterol, ApoB = Apolipoprotein B, ApoA-I = Apolipoprotein A-I, ApoB/ApoA = Ratio of Apolipoprotein B and Apolipoprotein A-I, S-Crea = Serum Creatinine, CKD = Chronic Kidney Disease, CVD = Cardiovascular Disease, S-Fructosamine = Serum Fructosamine, S-Glucose = Serum Glucose.

The mean age in the study population was 57 years with a range from 2 to 97 years. The subjects with normal glucose tolerance were somewhat younger than subjects with T2D. Those with DiagnosedT2D had more often a history of cardiovascular disease compared to the group with normal glucose tolerance. A history of cancer was similar across the categories. The NewT2D group had similar clinical characteristics compared to the DiagnosedT2D group except for a lower frequency of history of cardiovascular diseases. Mean values of triglycerides, total cholesterol, ApoB and the ApoB/ApoA-I ratio increased and mean values of HDL-C and ApoA-I successively decreased from subjects with normal glucose levels to those with pre-diabetes and newly diagnosed type 2 diabetes.

### Diabetes-induced increases of markers of glycaemia

In the fasting state, fructosamine increased from an average of 2.03 mmol/L in subjects with normal glucose tolerance to 2.11 mmol/L in the prediabetes group and to 2.62 mmol/L in the NewT2D group. Similarly to fructosamine, HbA_1c_ increased from an average of 4.76% (29 mmol/mol) to 5.14% (33 mmol/mol) in the prediabetes group and to 6.93% (52 mmol/mol) in the NewT2D group. Neither for fructosamine nor HbA_1c_ did DiagnosedT2D markedly diverge from NewT2D. Concentrations were markedly higher for the T1D group compared to the groups with type 2 diabetes (2.93 mmol/L for fructosamine and 7.62% for HbA_1c_).

### Associations between various glucose markers

**Figure 1:**
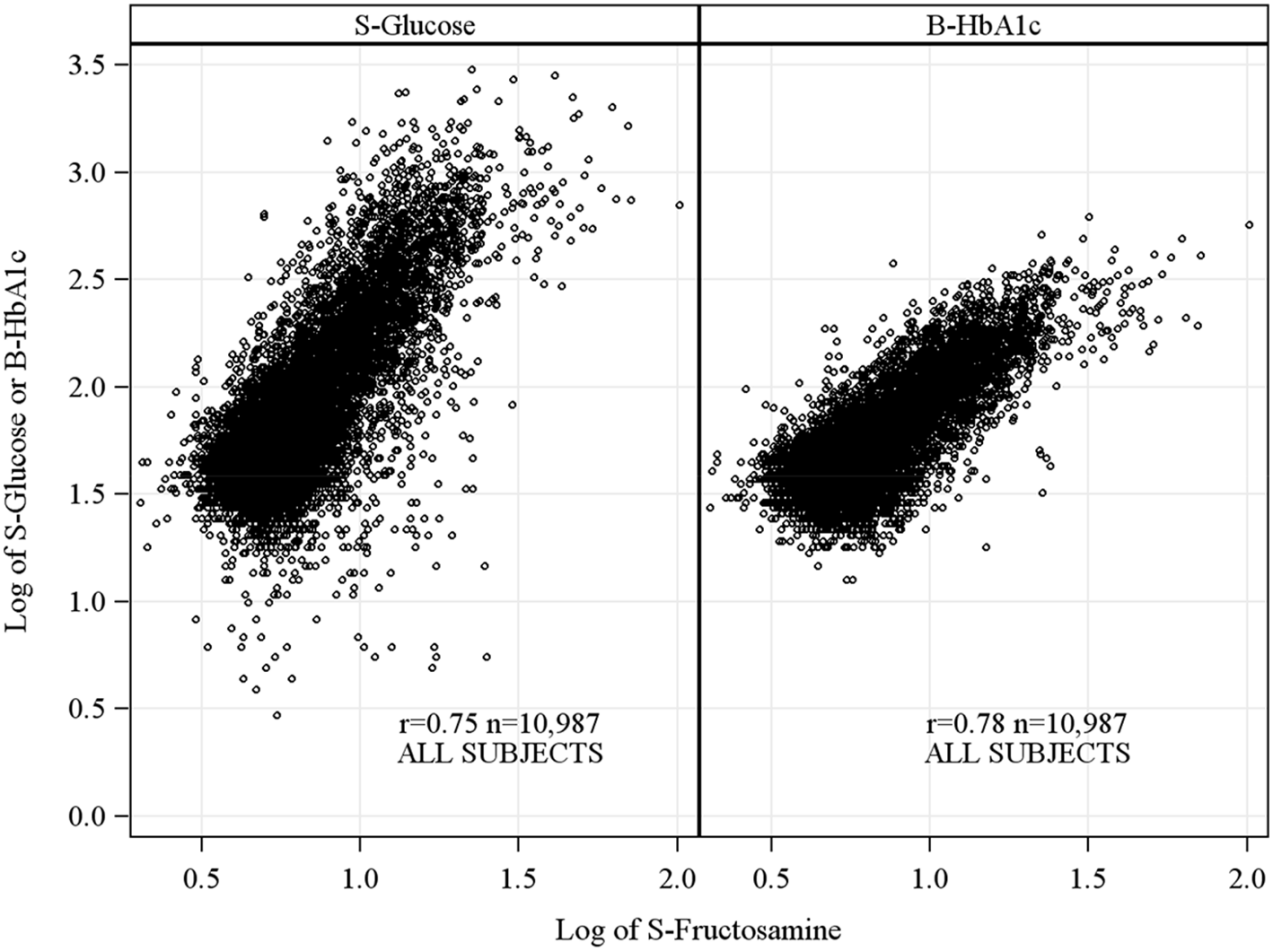
Overall associations of S-Fructosamine with S-Glucose and B-HbA_1c_ respectively. All fasting states and all levels of glycemia included (All subjects).

**Figure 2:**
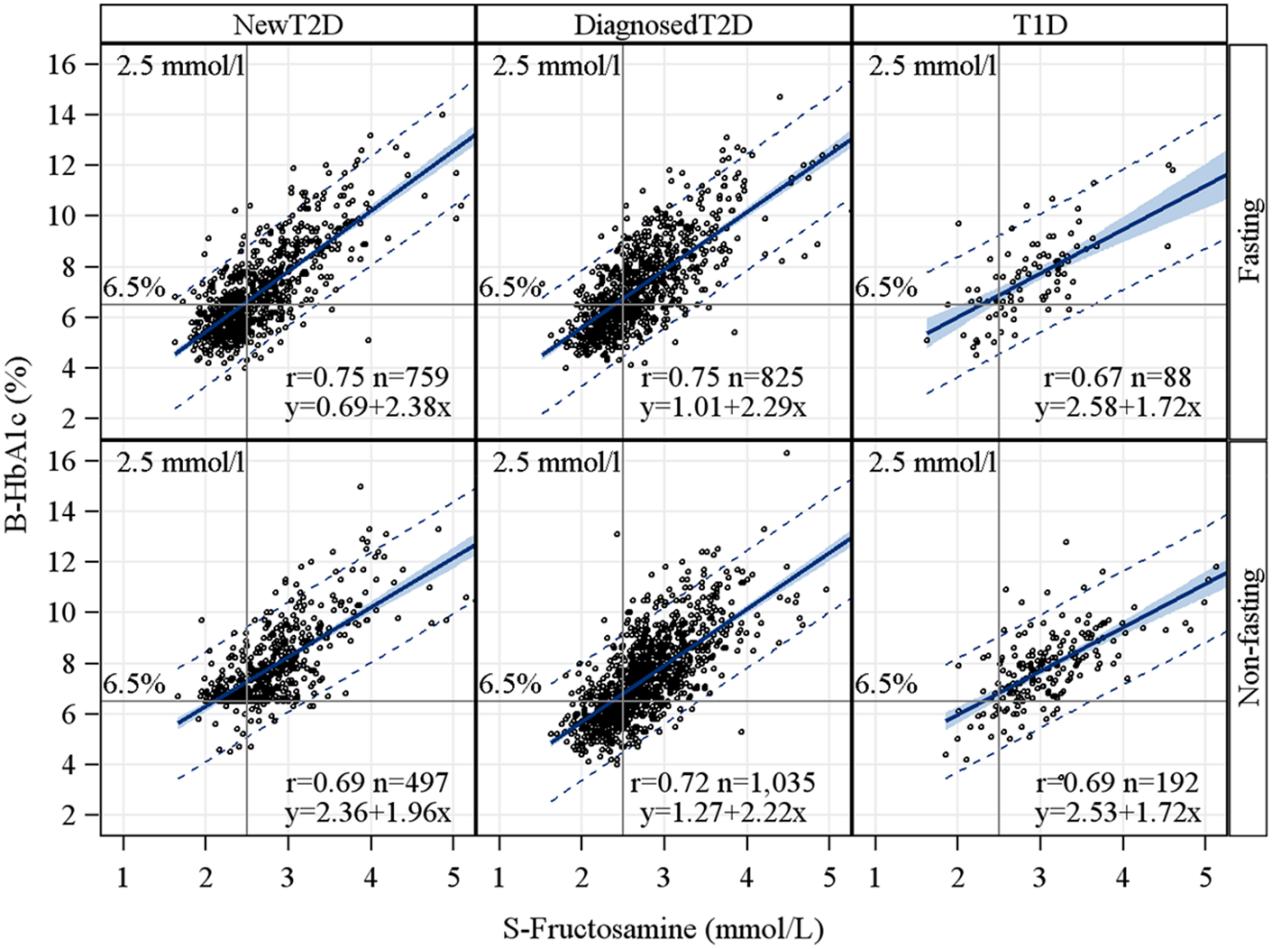
Correlations of S-Fructosamine and B-HbA_1c_ by fasting status, type 2 diabetes (new and diagnosed) and type 1 diabetes. Fitted regression line with 95% confidence and prediction limits. Reference lines at fructosamine 2.5****mmol/L and at HbA_1c_ 6.5% (48****mmol/mol).

Across glucose levels, strong associations were found for fructosamine with glucose and HbA_1c_ respectively, r = 0.75 and r = 0.78 respectively in the entire cohort (Figure 1). Fructosamine correlated with similar strength to HbA_1c_, r = 0.79 and glucose, r = 0.80 in the fasting state. The linear correlations for fructosamine and HbA_1c_ was identical, r = 0.75, in the NewT2D and DiagnosedT2D group respectively (Figure 2). Corresponding linear correlations for non-fasting subjects were r = 0.69 and r = 0.72. Subjects with T1D showed a similar association between fructosamine and HbA_1c_ as the subjects with DiagnosedT2D or NewT2D. The linear correlation was r = 0.67 in fasting subjects with T1D. Much lower correlations were found in the prediabetes group, r = 0.11 and r = 0.17, in the fasting and non-fasting subjects respectively and no clear association in subjects with normal glucose tolerance. Adjusting for gender, age, albumin, BMI, total cholesterol and triglycerides, affected associations only marginally. Exclusion of subjects with previously diagnosed anemia, chronic kidney insufficiency or diagnosed nephrosis respectively did not alter the associations.

### Fructosamine, HbA_1c_ and glucose in repeated measurements

**Figure 3:**
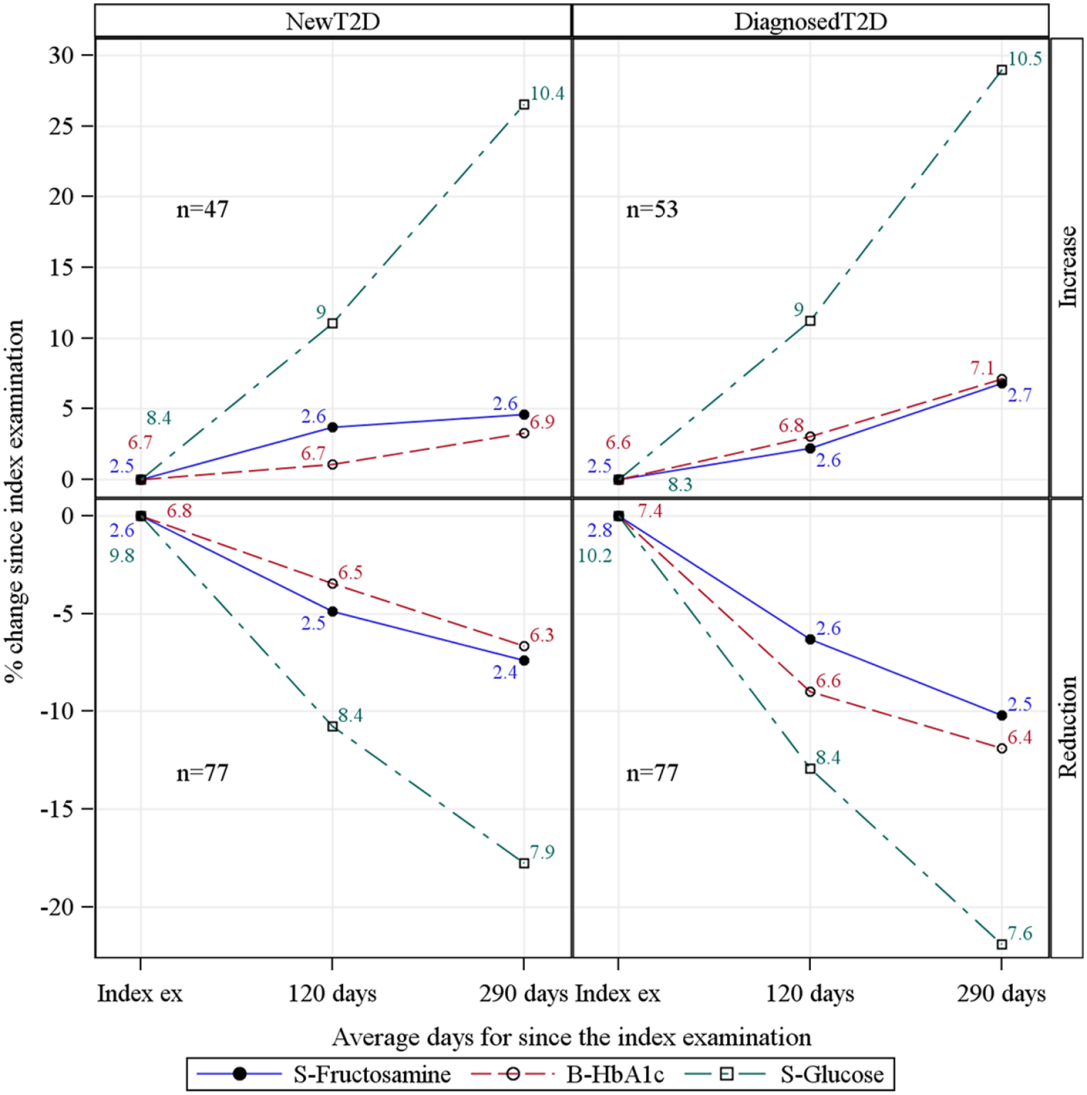
Mean values of S-Fructosamine, S-Glucose and B-HbA1c over time by increase/reduction of S-Glucose at on average 290 days after the index examination. Fasting subjects with new or diagnosed type 2 diabetes and with three simultaneous measurements of the biomarkers within one year are included.

**Figure 4:**
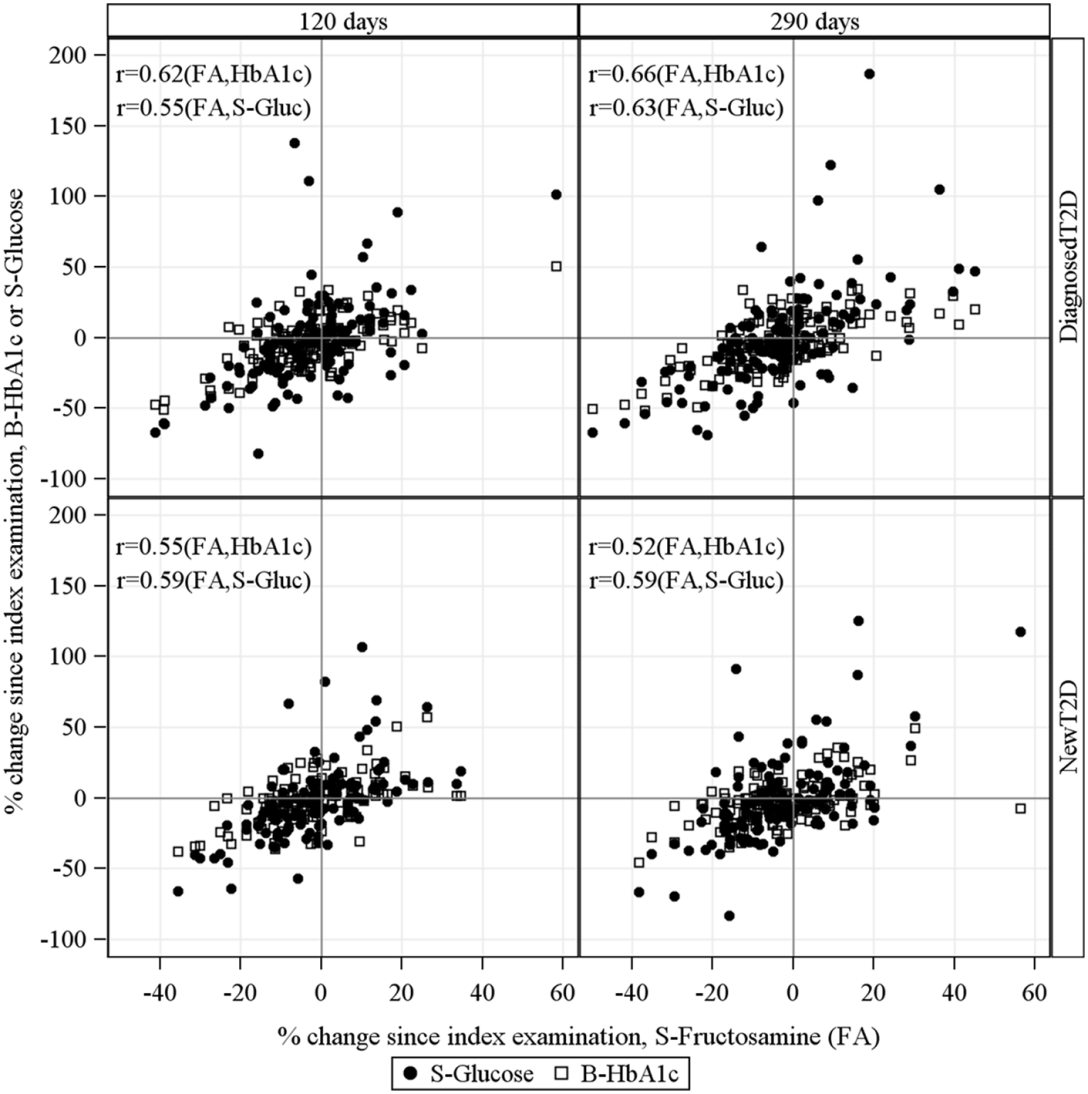
Individual changes and correlations of S-Fructosamine, S-Glucose and B-HbA1c over time. Fasting subjects with new or diagnosed type 2 diabetes and with three simultaneous measurements of the biomarkers within one year are included.

Figure 3 shows the results for fasting subjects with type 2 diabetes (n = 254) in whom three repeated measurements of glucose, fructosamine and HbA_1c_ were available. In the NewT2D group glucose levels had increased on average by 27% after a mean of 290 days. Simultaneously fructosamine and HbA_1c_ increased by 4.6% and 3.3%, respectively. Corresponding results for DiagnosedT2D subjects were a 29% increase in glucose, a 6.8% increase in fructosamine and a 7.1% increase in HbA_1c_. In subjects with NewT2D who manifested a decrease of mean glucose level by 18% after a mean of 290 days, the mean decrease of fructosamine was 7.4% and that of HbA_1c_ 6.6%. Corresponding results for the DiagnosedT2D group were for glucose 22%, fructosamine 10.2% and HbA_1c_ 11.9%. Thus, there were parallel changes over time in the evolution of glucose, fructosamine and HbA_1c_ in those who increased, and those who decreased their glucose level. Figure 4 shows individual changes in the biomarkers over time. The correlation coefficients for the changes were similar regardless of time point for follow-up.

### Hyperglycemia (diabetes) based on fructosamine compared with ADA diagnostic criteria

**Figure 5:**
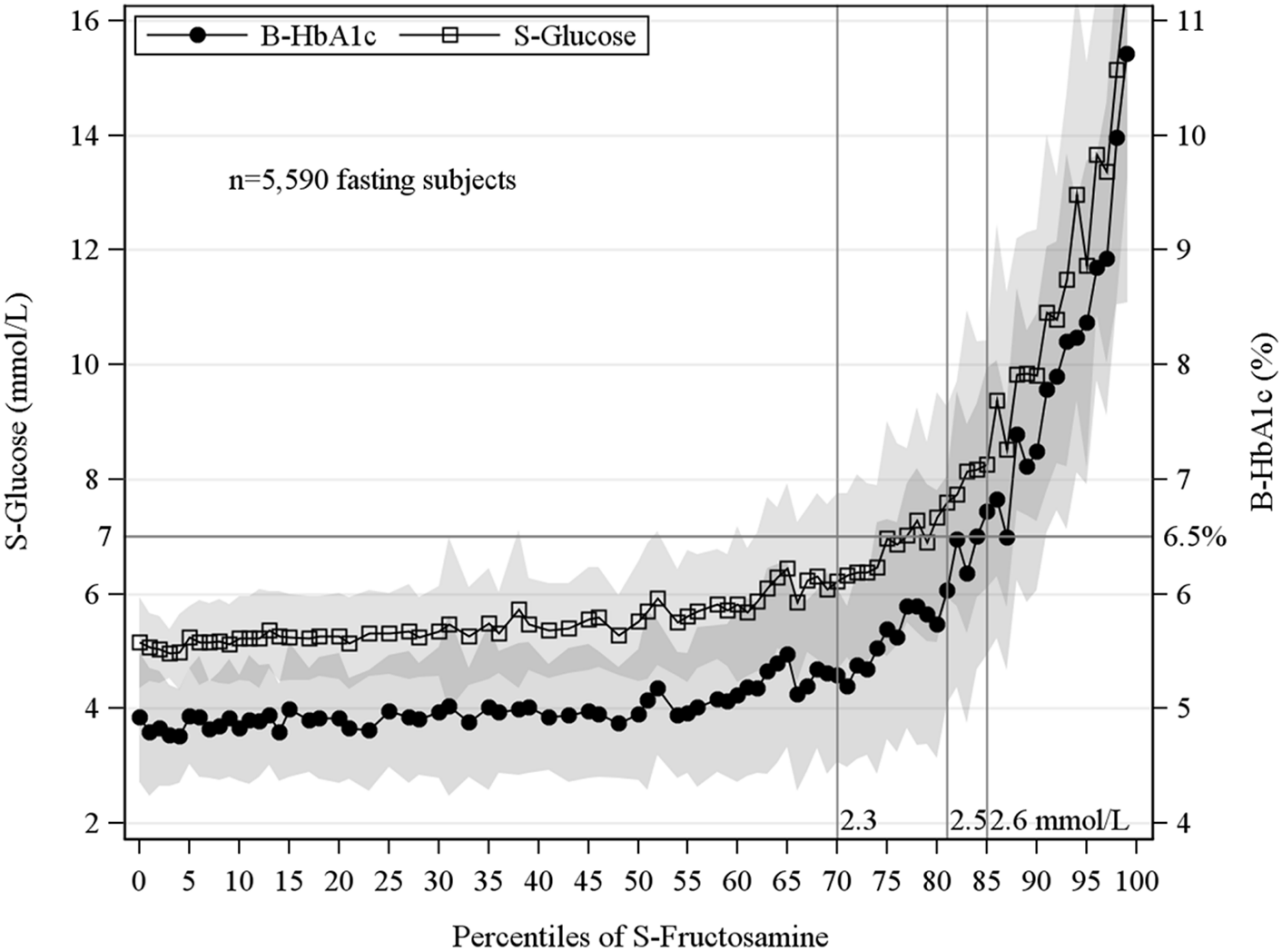
Mean values of fasting serum glucose and HbA_1c_ by percentiles of S-Fructosamine with corresponding 1 standard deviation. Sensitivity/Specificity at various fructosamine cut-off values for diagnosis of diabetes according to ADA criteria, 2.3 mmol/L (79%, 88%), 2.5 mmol/L (61%, 97%), 2.6 mmol/L (52%, 99%). Diagnostic discrimination of diabetes by S-Fructosamine using ROC analysis: AUC = 0.91 (fasting) AUC = 0.95 (non-fasting).

Mean values were calculated for HbA_1c_ and glucose respectively among the fasting subjects by percentiles of fructosamine (Figure 5). At a level of fructosamine of 2.5 mmol/L, the mean values of both glucose and HbA_1c_ were diagnostic for diabetes by ADA criteria. When ADA criteria for classification of diabetes, i.e. FSG≥7 mmol/L or an HbA_1c_≥6.5% (48 mmol/mol) was used a fructosamine cut-off of 2.5 mmol/L correctly diagnosed 61% as diabetes and correctly rejected 97% of the non-diabetes individuals. Corresponding sensitivity and specificity for non-fasting subjects were 82% and 94% respectively. The area under the ROC curve (AUC) for diagnosing diabetes by fructosamine when the ADA criteria were used to define diabetes was AUC = 0.91 in the fasting and AUC = 0.95 in the non-fasting state, a finding which indicated good discrimination across levels of fructosamine. By separately modelling HbA_1c_ and fructosamine respectively to explain diabetes defined solely based on glucose criterion, the AUCs were similar for the two biomarkers fructosamine and HbA_1c_, 0.91 and 0.90 respectively. The AUC obtained by defining diabetes by fructosamine when both glucose and HbA_1c_ criteria were met was AUC = 0.96.The fructosamine cut-off 2.5 mmol/L was applied in analyses of all fasting subjects in the AMORIS cohort, n∼255,000, using solely the glucose ≥7 mmol/L as criterion. The results showed identical sensitivity and somewhat higher specificity (61% and 98%) compared with the current study population (results not shown).

## Discussion

The present results are based on a large population representative for the working general population of Stockholm at the time of laboratory examinations. We found close associations between fructosamine, glucose and HbA_1c_, overall and particularly in subjects with either type 1 or type 2 diabetes. These findings are consistent with earlier but smaller studies [9]–[13] and also with recently published results from the ARIC study where an overall correlation of r = 0.82 was found [15]. Importantly, we found similar relations between fructosamine and HbA_1c_ irrespective of fasting or non-fasting. The inter-relationship between fructosamine and HbA_1c_ as well as association with glucose levels in those with prediabetes was less striking and was not seen in those with normal glucose values. Hence, the relationship between fructosamine and HbA_1c_ to fasting glucose levels respectively indicates a curvilinear relationship as has also previously been pointed out [7], [12]. Importantly, our results from AMORIS were obtained in a much larger population of men and women in all ages and with a much wider range of glucose (up to 40 mmol/L), fructosamine (up to 7.5 mmol/L) and HbA_1c_ (up to 16.5%), than in studies published previously [9]–[14]. Subjects with prediabetes and especially manifest diabetes were characterized by features common in subjects with metabolic syndrome and manifest diabetes such as high BMI, hypertriglyceridemia, low HDL cholesterol high apoB and low apoA-I giving the typical manifestations of high atherogenic apoB/apoA-I ratios as reviewed [27]. However, the relations between glucose, fructosamine and HbA_1c_ remained basically unchanged when controlling for age, gender, BMI or lipid levels. In addition, albumin correction of the fructosamine may only improve precision to a small degree regarding monitoring of variability of glucose values. Hence, the cross-sectional association of fructosamine with different degrees of hyperglycaemia appears robust.

Previously, Baker et al. showed strong association between fructosamine and HbA_1c_ in the interval r = 0.70 to r = 0.87 in patients with type 2 diabetes [9]–[11]. A more recent study by Cohen et al. included 153 subjects with varied types of diabetes [13]. They observed a correlation of r = 0.78. Juraschek et al. also reported a similar linear correlation but also suggested polynomial models as the best fit [12]. All of the above studies had rather small study populations with limited number of subjects with high glucose levels. Thus, the present AMORIS study confirmed the correlations from earlier work but in a much larger study population.

Moreover, Juraschek et al. evaluated various criteria for diabetes by presenting AUC measures. They reported an AUC of 0.83 for both fructosamine and fasting glucose if diabetes was defined by an HbA_1c_ of 6.5% (48 mmol/L) [12]. The diagnostic discrimination by using fructosamine in the AMORIS study, which is based on a much larger population, was higher, up to AUC = 0.95.

When subgroups of individuals in which glucose, fructosamine and HbA_1c_ were measured simultaneously up to 6 and 12 months (on average 120 and 290 days respectively), fructosamine and HbA_1c_ changed in parallel both in those who increased their glucose values and in those whose glucose values were considerably reduced (possibly due to better adherence to dietary and/or pharmacological therapy). Although individual discordant differences in directions were observed the major part of those was in individuals with minor changes of hyperglycaemia which likely would be more sensitive to random variation. Thus, these data indicate that fructosamine can be used to monitor glucose control over time in a similar way as HbA_1c_.

The results of the present study indicate that fructosamine discriminates well between subjects with and without diabetes across all levels of the biomarker with an AUC over 90%. For specific levels of fructosamine it is also possible to identify subjects with type 2 diabetes with a reasonable sensitivity and a high specificity. Our results would suggest a fructosamine somewhere in the range of 2.3–2.6 for this purpose. Recently, Selvin et al showed an adjusted hazard ratio for incident diabetes of 4.96 for those above 2.64 mmol/L compared to those below 2.41 mmol/L [15]. These levels are in line with those of our study. Notably, in follow-up studies of the outcome in diabetes patients a non-differential misclassification of exposure (in this case type 2 diabetes) in terms of reduced sensitivity does not influence estimates of relative risks. A reduced specificity would tend to bias relative risk estimates towards the null. For estimates of prevalence rates, and in particular in a clinical setting, the effects of a reduced sensitivity and specificity could be misleading or unacceptable. In our estimates of sensitivity and specificity we used diagnostic criteria based on fasting serum glucose and HbA_1c_ according to ADA guidelines. It cannot be ruled out that this ‘gold standard’ also misclassified some subjects. Thus, the interpretation of our findings should be made with the awareness of a certain degree of misclassification of the disease in all definitions based on the different biomarkers. The results of this study indicate that fructosamine does very well in the comparison to the established diagnostic biomarkers both in the identification of subjects with hyperglycaemia or diabetes and in the evaluation of long term glucose control in subjects with manifest diabetes. It would be of greatest importance to examine the prognostic association between fructosamine and major outcomes including cardiovascular disease and hence subsequent studies from the AMORIS cohort will have that focus.

### Strengths

Strengths of this study include the fact that a single laboratory was used for determination of all biomarkers (CALAB laboratories, Stockholm, Sweden), that all analyses were performed on fresh blood samples, that all methods are well documented and that they are rigorously standardized. It is therefore unlikely that the relations between these three markers of glucose metabolism are biased due to poorly controlled methods or erroneous classification or due to the variation of nutritional status and potential confounders in this large population. Further, the study’s size with 5,590 subjects known to be fasting whereof 3,063 with impaired or abnormal glucose levels, to our knowledge is the largest used for this purpose. Moreover it is a rather homogenous population with 80% known to be born in Sweden and additional 5% in Finland. The large AMORIS population of 812,073 men and women is representative of the larger Stockholm population as previously documented [28]. The sub-cohort studied in this paper is also representative for the whole AMORIS study population regarding education and country of birth but with a somewhat lower proportion of blue-collar workers.

### Limitations

The study population was required to have glucose, fructosamine and HbA_1c_ measured at the same time. It is likely that the individuals included in the study visited general physicians for evaluation of a possible diabetes diagnosis or for diabetes control. This could explain the fact that the proportion of subjects with type 2 diabetes in this study was much higher than in the general population. It is also of note that the proportion of individuals who had a history of cardiovascular diseases and cancer was higher in the study population compared with the whole AMORIS cohort.

We used a randomly measured glucose value of 11.1 mmol/L or above as a diagnosis of diabetes in non-fasting subjects. Although not strictly adherent to ADA criteria (to be used only for symptomatic hyperglycaemia), the small number diagnosed solely by this criterion in this study, n = 14, most likely did not affect the results. In addition, the AMORIS population consists largely of subjects born in Sweden or in other Northern European countries. With regard to age there is a broad representation including also many subjects less than 30 years of age.

In conclusion, fructosamine is closely associated with HbA_1c_ and glucose particularly in patients with type 1 or type 2 diabetes. Fructosamine and HbA_1c_ tend to parallel each other over time in subjects with diabetes indicating similar reflections of glucose control. The results also suggest that fructosamine discriminates well between subjects with and without diabetes. Fructosamine may be a useful biomarker of hyperglycaemia in epidemiological and clinical studies. Future outcome studies may investigate relationships between levels of fructosamine and risks to develop myocardial infarction, stroke and diabetic complications.
